# Forward steps, lingering gaps: gender representation among distinguished speakers at professional conferences

**DOI:** 10.1093/biosci/biaf063

**Published:** 2025-06-30

**Authors:** Sara Bombaci, Cooper Farr, Liba Pejchar, Kate D Wilkins, Tessa Wittman

**Affiliations:** Department of Fish, Wildlife, and Conservation Biology and with the Graduate Degree Program in Ecology, Colorado State University, Fort Collins, Colorado, United States; Conservation Science Program, Tracy Aviary, Salt Lake City, Utah, United States; Department of Fish, Wildlife, and Conservation Biology and with the Graduate Degree Program in Ecology, Colorado State University, Fort Collins, Colorado, United States; Department of Field Conservation, Denver Zoo Conservation Alliance, Denver, Colorado, United States; Ruckelshaus Institute, Haub School of Environment and Natural Resources, University of Wyoming, Laramie, Wyoming, United States

**Keywords:** gender equity, keynote address, plenary speaker, virtual meetings, women in science

## Abstract

Plenary speakers serve as role models for early-career scholars, and these talks advance the speakers’ careers while celebrating the important scientific contributions of women. Professional conferences are an ideal venue for assessing progress toward equity goals across disciplines. We examined gender disparities among distinguished speakers at North American ecology conferences from 2000 to 2023. We found that women's representation as speakers has increased, especially in the past decade, but they remain underrepresented relative to their proportion among graduate students. Disparities persist across institutions, disciplines, and career stages, particularly for women in later career stages. In addition, the COVID-19 pandemic did not notably affect women's representation, and nonbinary genders remain minimally represented, underscoring ongoing challenges in achieving inclusive representation. Although the gender gap has not yet closed, this emerging positive trend for women speakers at professional ecology conferences is encouraging.

Discourse about equity and social justice has increased among scholars and throughout society in recent years (Joseph and Hirshfield 2011, Toraif et al. 2023). However, it is unclear whether these conversations have changed how we value and reward the work of scientists and practitioners who have been historically excluded in science, technology, engineering, and math (STEM) disciplines. Past social movements and changing societal views have been important for advancing equity for disenfranchised groups (Louis 2009, Johnson 2013, Uluğ and Acar 2018), but this progress can be slow and is hindered by structural forces that create systemic injustice (McKeown 2021). For example, in the United States, the women's movement of the 1970s resulted in legislation, such as the Equal Pay Act (Elisburg 1978), to combat discriminatory practices. Despite some progress, those legal protections have not resulted in equal pay across genders (Baker et al. 2019).

Women are historically underrepresented in STEM fields, a disparity that persists despite heightened awareness of the importance of gender equity and efforts to improve representation of women in scientific fields (Debarre et al. 2018, Shannon et al. 2019). Gender equity in the sciences and across sectors results in increased productivity and employee retention, as well as improving innovation and decision-making (Potvin et al. 2018, Shannon et al. 2019). As of 2019, women represented less than 30% of scientific researchers globally, and only 32% of research scientists in North America were women (Shannon et al. 2019). Participation in academic societies as a member of the board, a presenter, or a speaker positively affects a scientist's career development, networking, and promotion potential, but women constitute only approximately 30% of leadership roles in academic societies (Potvin et al. 2018). Equal representation of women in societies tends to result in increased representation of women in scientific society leadership roles and as speakers at conferences (Isbell et al. 2012, Debarre et al. 2018, Potvin et al. 2018).

Professional conferences are an ideal venue for assessing progress toward equity goals within and across disciplines. In recent years, professional societies have attempted to make conferences more representative of an increasingly diverse population (Lerman et al. 2021), including highlighting the accomplishments of women through awards and distinguished talks (Sarabipour et al. 2020). Plenary speakers play an outsized role at conferences, because these speakers can serve as role models for the next generation of scholars and change makers (Downing et al. 2005). Furthermore, giving a plenary talk or serving on a distinguished panel is an important mark of achievement (Kalejta and Palmenberg 2017) and is valued in tenure and promotion decisions (Debarre et al. 2018, Singhal et al. 2020).

Our previous study, which documented a gender gap among distinguished speakers at North American ecology conferences that took place from 2000 to 2015 (Farr et al. 2017), offers an opportunity to evaluate whether national discourse on equity and social justice has since closed the gender gap. In that study, we found that women constituted only 15%–35% of distinguished speakers within the conferences we examined, with no consistent pattern of increasing representation as speakers over that 15-year period (Farr et al. 2017). Within our dataset, distinguished speakers in later career stages and working in the private sector were significantly less likely to be women. The patterns varied within the subdiscipline of ecology, with fields such as animal behavior and restoration ecology emerging as the fields with the highest representation of women as distinguished speakers. The 9 years that have passed since our previous study have seen a growing awareness around issues related to diversity, equity, and inclusion within STEM disciplines (Ali et al. 2021, Singleton et al. 2021); gender representation in invited speakers (Sardelis et al. 2017, Shillcutt and Lorenzen 2020); and significant societal shifts related to the COVID-19 pandemic (hereafter, *COVID*; Niner et al. 2020).

Our goals in this study were to assess whether the previously documented gender disparity among distinguished speakers at professional ecological conferences in North America persists (Farr et al. 2017) and to evaluate which factors influence speaker gender ratios. Specifically, our objectives were to evaluate potential changes in gender ratios among conference speakers across more than two decades (2000–2023); to identify whether the combination of events beginning in 2020, including the COVID pandemic, move to virtual conferences, and concurrent social justice movements in North America are associated with shifts in speaker gender ratios; and to determine which characteristics of speakers and their institutions (i.e., career stage, field of expertise, institution type, year) influenced the probability that speakers were women.

Although we are not testing causal relationships with this study, we developed a series of predictions grounded in the existing literature and a theoretical understanding of gender-based inequities during this time. To understand whether the plenary speakers reflected graduate student demographics and the potential for speakers to serve as role models, we compared the proportion of women in distinguished speaker roles at ecology conferences (2000–2023) with the proportion of women graduate students in the field of ecology (2000–2021). We predicted that the proportion of women distinguished speakers would continue to be lower than that of the graduate student population but would have increased over time because of heightened awareness of identity-based inequities within STEM disciplines and social injustices within society (Joseph and Hirshfield 2023, Toraif et al. 2023). In addition, we assessed whether the proportion of women as distinguished speakers corresponded to the overall proportion of women ecologists in early-, mid-, and late-career stages in academia. On the basis of findings from Farr and colleagues (2017), we predicted that the proportion of women distinguished speakers would match that of early- to midcareer professionals but exceed that of late-career professionals. We also predicted that there would be a lower representation of women speakers after COVID, given that the pandemic was found to disproportionately affect women in caregiver roles (Aubry et al. 2021). Furthermore, we predicted that speaker characteristics such as career stage, affiliated institution type, and field of expertise would be associated with the probability that a speaker was a woman (Farr et al. 2017). For example, we expected that women in late career stages would be less likely to be distinguished speakers, because of ongoing barriers women face in advancing to full professor or equivalent position in the sciences (Farr et al. 2017, Cardel et al. 2020).

## Assessing gender representation among distinguished speakers

To assess gender representation among distinguished speakers, we followed the methods outlined in Farr and colleagues (2017). We defined distinguished speakers as plenary and keynote speakers, award recipients, and panelists (hereafter, *distinguished speakers*). We collected information on distinguished speakers from the same list of societies and associated conferences (*n* = 70 societies, 249 conferences; supplemental file S1, table S1) listed in (Farr et al. 2017), with the exception of a new conference hosted by the Society for Urban Ecology. All conferences on the list took place in North America between 2000 and 2015 (Farr et al. 2017) and between 2016 and 2023 for the new data set. We collected the following data for each conference: the society hosting the conference; the conference title, society subfield (supplemental file S1 table S2), and year; whether the conference was in person, virtual, or hybrid; speaker names, titles, career stage, field of expertise, and pronouns; what type of talk the speaker gave (i.e., award recipient, keynote, panelist or moderator, or plenary); the speaker's organization; and the type of institution with which the speaker was affiliated (supplemental file S1 table S3). The career stages were defined using titles and years of postdoctoral experience. Early-career professionals were speakers with titles that included *assistant* or *junior* or those with 0–7 years of postdoctoral experience. Intermediate-career professionals were speakers with titles that included *associate* or those with 8–14 years of postdoctoral experience. Late-career professionals were speakers with titles that included *full, director*, or *senior* or those with more than 15 years of postdoctoral experience. We collected pronouns for each speaker from their speaker biography. In the present article, we refer to speakers with *she/her* pronouns as women, those with *he/him* pronouns as men, and speakers with *they/them* pronouns as gender nonbinary. The number of speakers identifying as nonbinary (*n* = 1) was extremely limited; therefore, we were unable to include nonbinary speakers in our statistical analyses.

We compared gender balance among early-career ecologists in academia with that of the conference speakers from our data set (2000–2023) by collecting multiple years of gender data (2000–2021) in the field of ecology from the National Science Foundation's (NSF) Survey of Graduate Students and Postdoctorates in Science and Engineering (supplemental file S1 table S4). To calculate the proportion of women speakers at conferences (the data our team collected), we divided the number of speakers with *she/her* pronouns by the total number of speakers (excluding speakers where we could not find pronouns listed). The data NSF collected used the term *female*, which we used to calculate the proportion of women graduate students in ecology (i.e., the number of female graduate students in ecology divided by the total number of graduate students in ecology) for each year. Next, to compare the gender balance of the conference speakers with that of professors across different career stages, we used conference speaker data from 2020 and data collected in 2020 by Jensen and Bombaci (2024) on the proportion of women in the ecological and environmental sciences who were full, associate, or assistant professors at US academic institutions. We tested the null hypothesis that the proportion of women distinguished speakers differed significantly from the proportion of women in full, associate, or assistant professor positions using a two-sample *z*-test (stats package prop.test function; v4.2.2; R Core Team 2023).

We tested the prediction that individual or institutional characteristics (i.e., career stage, field of expertise, institution type, year, and a binary predictor for whether the conference occurred before or after COVID) influenced the probability that a distinguished speaker was a woman. Our response variable was a binary indicator of whether a speaker was a woman (1) or man (0). To evaluate relationships between the response and predictor variables, we fit multiple generalized linear mixed-effects models to our data with a binomial distribution using glmer function in the lme4 package (version 1.1.35.1; Bates et al. 2015) in R (R version 4.3.0, R Core Team 2023). We also included a random effect for the conference subfield because multiple observations were recorded for each conference and there was overlap of many conferences across subfields (e.g., the American Ornithologists Union and the Cooper Ornithological Society had many joint conferences).

We used a multimodel inference approach (Burnham and Anderson 2002) and Akaike's information criterion to compare all possible combinations of models that included our predictor variables (career stage, field of expertise, institution type, year, and COVID) as additive effects. We chose to fit all possible combinations of our predictor variables because we hypothesized that any of these factors could affect the probability that a distinguished speaker was a woman, either independently or as additive effects with other predictors (see predictions in introduction). We used model selection based on the Akaike information criterion corrected for small sample sizes (AICc) using the dredge function from the MuMIn package in R (version 1.47.5; Bartoń 2023). First, we fit a full (global) model that included all predictor variables. Following this, we generated a set of all possible models, given the predictor variables in the full model, and ranked them according to their AICc values. We considered all models with ΔAICc < 4 to be well-supported models (Burnham and Anderson 2002) and based our interpretation on the estimates from these models. To assess the influence of the hypothesized predictors, we used our well-supported models to evaluate the direction of each model coefficient and whether the 95% confidence intervals surrounding the coefficient overlapped 1 (an odds ratio of 1 indicates that the characteristic is equally likely to influence the probability of a speaker being a woman or a man).

We also checked the assumptions of the models to avoid overfitting and to ensure the validity of the conclusions drawn from the model selection process. We used the testDisperson, testUniformity, and testOutliers functions in the car (version 3.1.2; Fox and Weisberg 2019) and DHARMa (version 0.4.6; Hartig 2022) packages, and we viewed diagnostic quantile–quantile plots and residual versus predicted values plots in R. Plots were created in R (version 1.47.5; R Core Team 2023) using built-in base functions, as well as functions from ggplot2 (version 3.3.2; Wickham 2016) and the SjPlot package (version 2.8.15; Ludecke 2023). In terms of model performance, the model diagnostics indicated good performance (supplemental file S1 figure S1). The nonparametric dispersion test did not show evidence of overdispersion for all four top models (*p* = .79, .80, .85, and .82 for the first through fourth best models, respectively). Likewise, the Kolmogorov–Smirnov test did not show evidence of a lack of uniformity for all four top models (*p* = .34, .30, .44, and .58 for the first through fourth best models, respectively), and there was no evidence of outliers (outlier test *p* = 1.0 for all four top models). Finally, the quantile–quantile residuals plots and the residual versus predicted values plots suggest that the data follows the expected distribution (supplemental file S1 figure S1).

## Gender representation among distinguished speakers at professional conferences

**Figure 1.**
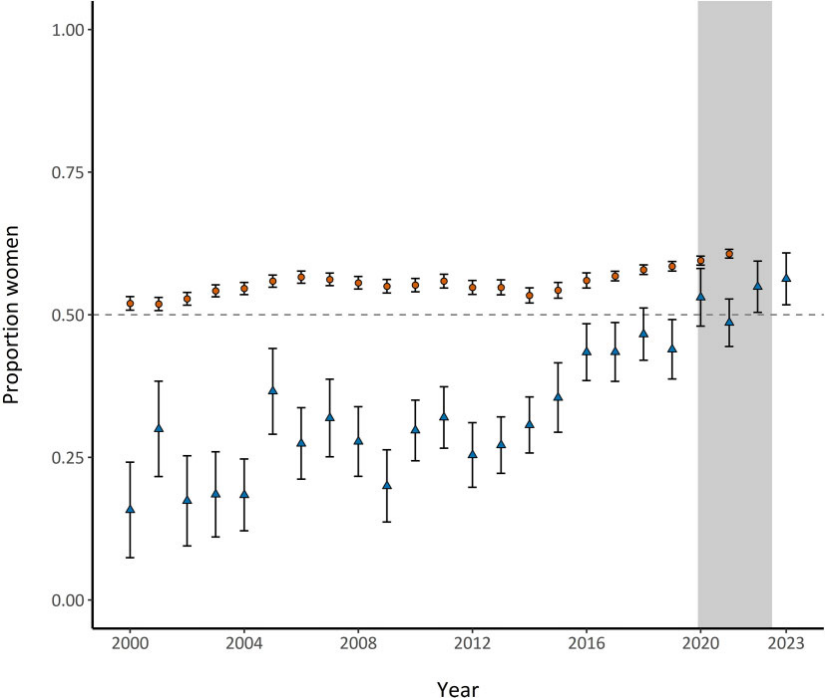
The proportion of distinguished speakers at ecology conferences with she/her pronouns from 2000 to 2023 (triangles; the data were derived from conference data; n = 1692) and the proportion of women graduate students in ecology from 2000 to 2021 (circles; the data were derived from the National Science Foundation and National Institutes of Health graduate data set; n = 49,189 speakers across all years and genders). The vertical gray bar represents the period after COVID 19 that also coincides with the rising social justice movement in 2020. The error bars represent the standard error. The dashed line is drawn at 0.50.

Our findings indicate recent progress in the representation of women in distinguished speaker roles at ecology conferences. The proportion of women distinguished speakers increased from 2000 to 2023, surpassing 50% for the first time in 2020 (figure 1). Although the proportion of women distinguished speakers was consistently below that of women graduate students across all years, the gap between these groups began to narrow in 2020 and has been diminishing, especially in the last 3 years (figure 1). This trend may reflect growing awareness of equity issues in academia (Stepan-Norris and Kerrissey 2016, Casad et al. 2021, Stockard et al. 2021) and active measures to address gender inequalities in speaking roles, such as including more women on selection committees (Martin 2014, Sardelis and Drew 2016) and implementing equitable selection policies (Vallence et al. 2019).

**Figure 2.**
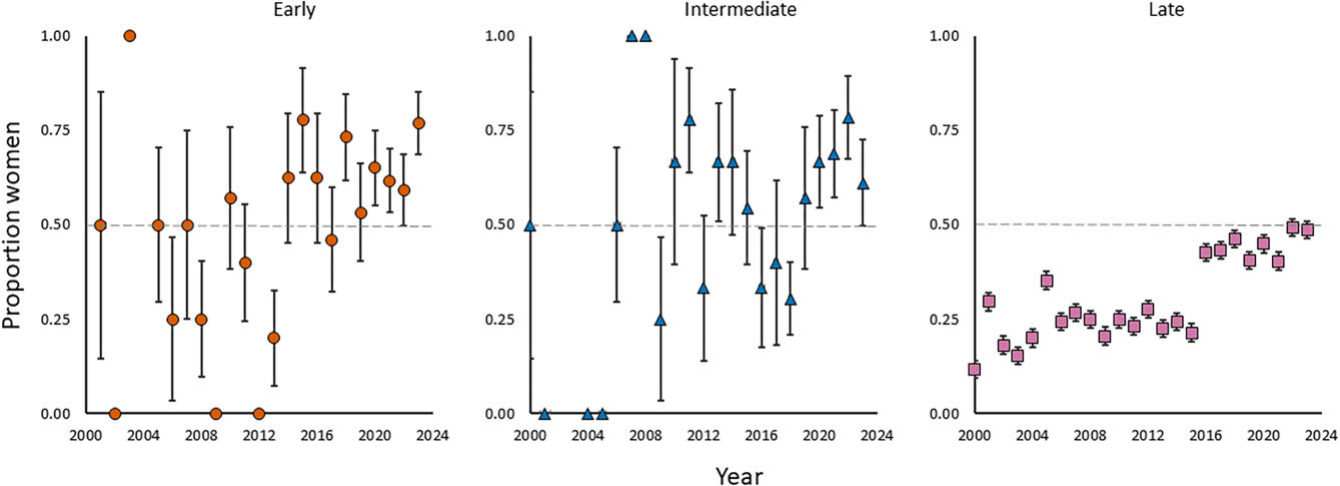
The proportion of distinguished speakers at ecology conferences with she/her pronouns from 2000 to 2023 across different career stages (early, n = 240; intermediate, n = 172; late, n = 1280; the sample size represents the total number of speakers across all years and genders for each category). The markers on the 0 line indicate that the proportion of women speakers for a given career stage was 0 for a given year. If no marker is present, then there were no speakers with she/her or he/him pronouns for a given career stage that year. The error bars represent the standard error.

**Figure 3.**
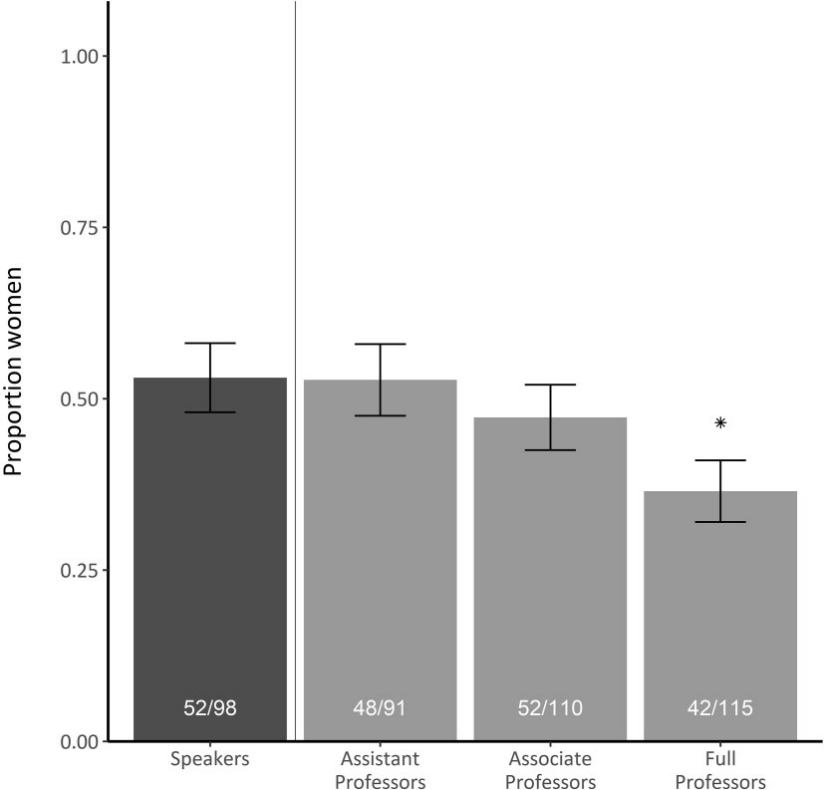
The proportion of distinguished speakers with she/her pronouns at ecology conferences in 2020 (the dark gray bar indicates data derived from the conference data set) and the proportion of women at different professional levels in ecology careers at academic institutions in 2020 (the light gray bars indicate data derived from a survey sent to ecology professors in October of 2020; Bombaci et al. 2024). The vertical line divides the separate data sets. The asterisk indicates that the proportion of women faculty significantly differs from the proportion of women distinguished speakers (p = .02; two-sample z-test of difference between proportions). The error bars represent the standard error.

**Figure 4.**
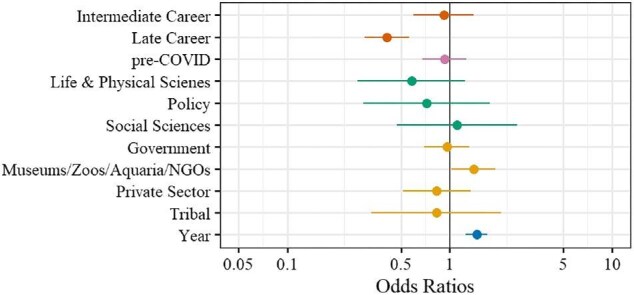
Odds ratios and 95% confidence intervals from the fourth best model of characteristics that influenced the probability of a distinguished speaker being a woman (the first, second, and third best models included a subset of the predictors in the fourth best model and coefficients and confidence intervals were similar across all models). Odds ratios are for variables of career stage (early, intermediate, and late), COVID (before or after COVID), field of expertise (arts and humanities, life and physical sciences, policy, and social sciences), institution type (academia; government; museums, zoos, aquaria, or nongovernmental organizations; private sector; tribal), and year. The categories of early career stage, post-COVID, arts and humanities, and academia are absorbed in the intercept, and ,therefore, the odds ratios for each variable are relative to those intercepts. An odds ratio of 1 indicates that the characteristic is equally likely to influence the probability of a speaker being a woman or a man. The odds ratios where 95% confidence intervals do not overlap 1 indicate that the characteristic is more likely to either positively or negatively influence the probability of a speaker being a woman.

Recent progress in representation of women speakers was not consistent across career stages. Although women in late career stages represented the largest total number of distinguished speakers, they also represented the lowest proportion among the other career stages (figure 2, supplemental file S1 table S5). In 2020, the proportion of women distinguished speakers was similar to the proportion of women associate and assistant professors but significantly higher than that of women full professors (figure 3). These trends were also evident in our models of relationships between speaker characteristics and the probability of a distinguished speaker being a woman. Our analysis resulted in four well-supported models (ΔAICc < 4; Burnham and Anderson 2002), with all models indicating a positive effect of year and a negative effect of late career stage (figure 4, supplemental file S1 table S6, supplemental file S1 figure S2). For each additional year, the odds of the speaker being a woman was 1.5 times more likely (figure 4). Speakers in late career stages were 44% less likely (1 – exp(–0.58)) to be women than were speakers in early and intermediate career stages, holding other variables constant (figure 4).

Our findings show that early- and intermediate-career women have been well represented as distinguished speakers, often at or above parity with men (figure 2). This may reflect broader gender shifts in academia (Spoon et al. 2023) and greater diversity considerations in speaker selection for these career stages. Despite these findings and the recent positive trends, women remain underrepresented among speakers in late career stages, likely because of overall lower representation of women in these stages (figures 2–4). This disparity highlights persistent challenges women face in advancing through academic and scientific careers, aligning with studies that show underrepresentation of women in faculty roles across fields despite high rates of graduate degree attainment (Wapman et al. 2022, Spoon et al. 2023). A recent survey of 245,270 US tenure-track and tenured professors revealed that women faculty across all academic fields face a heightened risk of leaving academia (Spoon et al. 2023). Specifically, women were 6%, 10%, and 19% more likely than men to leave each year at the assistant, associate, and full professor levels, respectively (Spoon et al. 2023). This disparity persists even after controlling for factors such as faculty career age, employer prestige, and whether professors received their training in the US or abroad (Spoon et al. 2023).

The term *leaky pipeline*, originally coined to describe the phenomenon by which women drop out of academic and scientific careers before reaching advanced positions, may not fully capture the active nature of the systemic barriers faced by women who are forced to leave their careers (Pell 1996). These barriers, which include the unequal distribution of parenthood pressures (Cech and Blair-Loy 2019), lower salaries (Martinez et al. 2017), and workplace dissatisfaction (Gardner 2012), disproportionately affect women. A comprehensive study across United States–based PhD-granting departments showed that women, especially those at the full professor level, were less likely to be promoted and more likely to feel pushed out of their jobs than men, with workplace climate often cited as a primary reason for leaving positions (Spoon et al. 2023). Furthermore, research indicates that women in academia tend to contribute more effort but receive less recognition, such as authorship, awards, and professional advancement opportunities (Feldon et al. 2017, Sarsons 2017, Vaughan et al. 2019, Ross et al. 2022). Consistent with these findings, we observed notable differences in women's representation across different types of talks. Women speakers had greater representation as moderators or panelists (50%) and lower representation in potentially more esteemed speaking roles such as keynote speakers (43%), award recipients (41%), and plenary speakers (36%) (supplemental file S1 table S7). Addressing these disparities in contribution, attribution, and representation is critical for advancing the professional careers of women scientists. Our research includes a large and diverse list of ecological societies with varying inclusivity goals. Previous studies have found that when societies include women as leaders or organizers for conferences, as well as implement equality statements, the representation of women as presenters increases (Debarre et al. 2018, Potvin et al. 2018).

**Figure 5.**
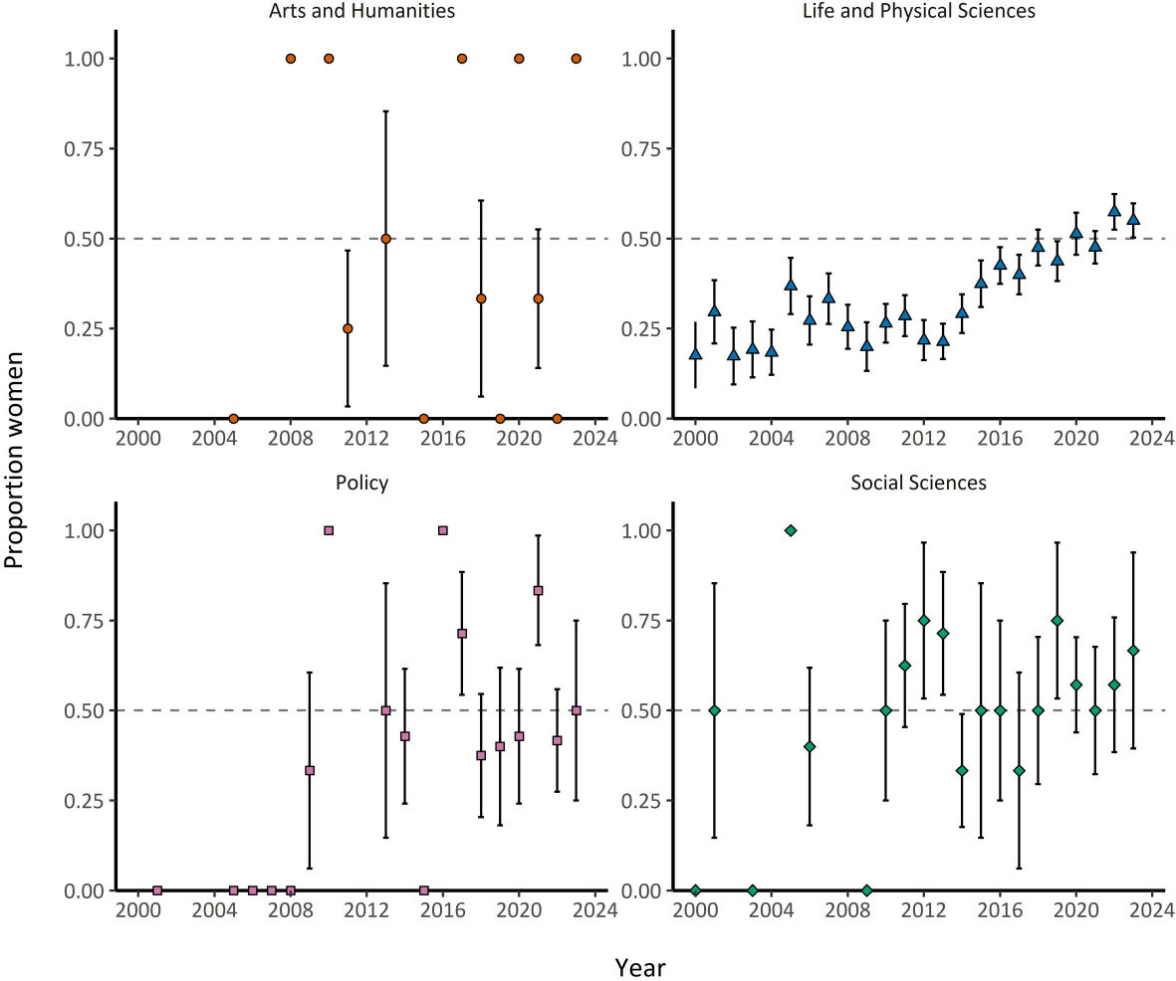
The proportion of distinguished speakers at ecology conferences with she/her pronouns from 2000 to 2023 across different fields of expertise (arts and humanities, n = 31; life and physical sciences, n = 1491; policy, n = 73; social sciences, n = 96; the sample size represents the total number of speakers across all years and genders for each category). The markers on the 0 line indicate that the proportion of women speakers for a given field of expertise was 0 for a given year. If no marker is present, then there were no speakers with she/her or he/him pronouns for a given field of expertise that year. The error bars represent the standard error.

**Figure 6.**
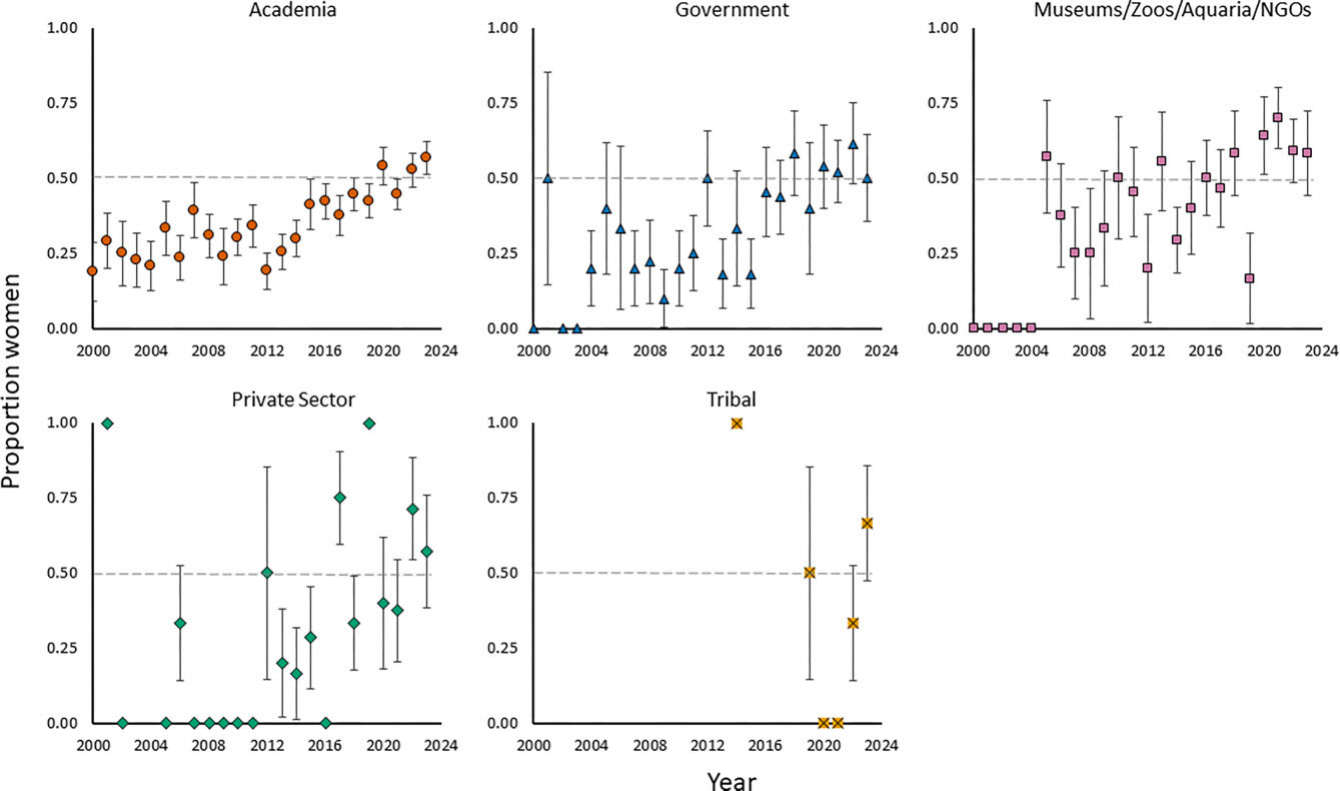
The proportion of distinguished speakers at ecology conferences with she/her pronouns from 2000 to 2023 across different institution types (academia, n = 1135; government, n = 223; museums, zoos, aquaria, or nongovernmental organizations, n = 220; private sector, n = 93; tribal institutions, n = 21; the sample size represents the total number of speakers across all years and genders for each category). The markers on the 0 line indicate that the proportion of women speakers for a given institution type was 0 for a given year. If no marker is present, then there were no speakers with she/her or he/him pronouns for a given institution type that year. The error bars represent the standard error.

Despite overall gains, women remain underrepresented as distinguished speakers in certain fields of expertise or institution types. Women were 32% (1 – exp(–0.38)) and 21% (1 – exp(–0.24)) less likely to be speakers in the life and physical sciences and policy, respectively, than in arts and humanities (reference group) and social sciences, although the confidence intervals overlapped 1 (figure 4, supplemental file S1 table S6, supplemental file S1 figure S2). The life and physical sciences had the most distinguished women speakers but the lowest percentage among the other fields (supplemental file S1 table S8), and representation of women as speakers in life and physical sciences was consistently lower over time, whereas arts and humanities, policy, and social sciences varied annually (figure 5). Therefore, despite recent positive trends in women speakers overall, women speakers in the life and physical sciences remain underrepresented relative to other fields. These disparities could be indicative of field-specific challenges and barriers that disproportionately affect women in the life and physical sciences. These include implicit biases that associate men with hard sciences and women with humanities, as well as stereotype threats—for example, negative stereotypes implying that women are less competent or capable in STEM fields than men are (Carli et al. 2016, Thébaud and Charles 2018, Light et al. 2022). With respect to institution type, speakers affiliated with museums, zoos, aquaria, or nongovernmental organizations were 1.4 times (40%) more likely to be women than were speakers in academia (reference group), but speakers in government, private sector, and tribal institutions were likely to be women at a similar rate to that of speakers in academia (figure 4; supplemental file S1 figure S2). Academia had the most distinguished women speakers, but the second lowest percentage among the other institutions, whereas museums, zoos, aquaria, and nongovernmental organizations had the highest percentage (supplemental file S1 table S9). The latter group consistently exceeded 50% representation from 2000–2023, unlike government, private sector, and tribal institutions (figure 6). This finding may be attributed to women being the majority of zoo staff, as well as students in zoo and aquaria graduate programs (Maple 2021). However, the proportion of women speakers from zoos and aquaria is still lower than the proportion of women working within these institutions (Maple 2021).

We also found that women's representation as distinguished speakers varied considerably across different subfields and societies in ecology and environmental science. In the subfields of animal behavior, marine biology, restoration ecology, botany, and general ecology, women speakers were represented at 44% or above, and the odds ratios were positive for these fields, whereas women were under 30% of speakers in the fields of range management, herpetology, ichthyology, and urban ecology, and the odds ratios were negative for most of these fields (supplemental file S1 figure S3, supplemental file S1 table S10). At the society level, the Association of Field Ornithologists, Society for Ecological Restoration, the Consortium of Aquatic Sciences, and the Society of Wetland Scientists had the highest representation of women speakers (at or above 50%), and societies such as the Society for Urban Ecology, the Society for the Study of Amphibians and Reptiles, the American Society of Ichthyologists and Herpetologists, the Society for Range Management, and the Weed Science Society of America had the lowest (at or below 20%; supplemental file S1 table S1). Some societies, such as the combined aquatic societies and the Animal Behavior Society, consistently maintained high representation of women speakers over time (supplemental file S1 figure S4). In contrast, many societies have shown notable increases in women's representation over the past decade, including the Society for Ecological Restoration, the Botanical Society of America, the Society of Integrative and Comparative Biology, the combined evolution societies, the International Association of Landscape Ecology, the Entomological Society of America, and the Raptor Research Foundation (supplemental file S1 figure S4). However, some societies with historically low representation of women, such as the Cooper Ornithological Society, the combined rangeland societies, and the combined herpetological societies, have remained persistently low (supplemental file S1 figure S4).

The higher representation of women speakers in fields such as marine biology and animal behavior may be attributed to various factors. Marine biology has evolved from a field that historically discriminated against women scientists, who were often barred from seafaring vessels because of superstition, faced harassment in the field, and encountered open discriminatory practices both in academia and the field (Legg et al. 2023). Currently, NSF data indicates that approximately 50% of PhDs in marine biology are awarded to women, but gender disparities persist in metrics such as professional advancement, leadership roles, and authorship (Burdett et al. 2022, Legg et al. 2023). In the field of animal behavior, women have surpassed parity in authorship, representing 58% of authors on identified papers published between 1966 and 2022; however, women are still underrepresented as lead authors (Gavriilidi and Van Damme 2023). Conversely, in herpetology, fewer than one in three authors were women in the last decade, and disparities persist for women in leadership roles (Rock et al. 2021). In mammalogy, women averaged around 32% of lead authorship from 1995 to 2016, but their representation in leadership roles and as award recipients in the American Society of Mammologists remains far below parity (Dizney et al. 2019). Similarly, the significant increases in women's representation as speakers observed in several societies over the past decade indicate that targeted efforts, such as diversity initiatives or policy changes, may be contributing to positive trends. However, the persistent underrepresentation of women speakers in certain societies, such as those focused on rangeland science and herpetology, suggests that systemic barriers may still exist in these fields, potentially requiring more intentional interventions to promote gender equity. These findings support our results and suggest that gender bias remains prevalent across many fields, particularly evident in the underrepresentation of women in leadership positions.

Contrary to our predictions, the COVID pandemic did not significantly affect the representation of women in distinguished speaking roles (7% less likely before COVID, but the confidence intervals overlapped 1; figure 4, supplemental file S1 table S6, supplemental file S1 figure S2). The evidence suggests that the pandemic exacerbated existing structural inequalities, such as the gendered distribution of childcare duties (Breuning et al. 2021, Stefanova et al. 2023), so we hypothesized that the pandemic would reduce women's ability to accept distinguished speaking invitations because they were disproportionately required to perform caregiver roles during this time (Aubry et al. 2021). However, the pandemic also led to an increase in virtual conferences, which may have mitigated some of the previous gendered barriers associated with travel for conferences (Sardelis et al. 2017, Niner et al. 2020). It is possible that these factors offset each other, resulting in no significant impact of the pandemic on women's representation as distinguished speakers.

## Forward steps, lingering gaps

Our results align with those of recent studies documenting positive trends in women's representation at scientific conferences. For example, Kalejta and Palmenberg (2017) found increasing representation of women among invited speakers at four prominent virology conferences, and Catalán and colleagues (2023) noted an increase in women plenary speakers at limnological conferences in recent years. However, despite this progress (Kalejta and Palmenberg 2017, Moeschler et al. 2019, Singhal et al. 2020, Catalán et al. 2023), these studies and others document lingering gender inequalities among invited speakers, particularly within certain fields (Nittrouer et al. 2018, Arora et al. 2020, Shillcutt and Lorenzen 2020). This pattern of variation across fields was also evident in our study, spanning multiple subfields of ecology. Although the gap is not yet closed, the emerging positive trend is significant. Distinguished speakers serve as role models for early-career ecologists and have the opportunity to highlight important scientific contributions they are making at high levels in their fields, which could lead to more career advancement prospects (Downing et al. 2005, Drury et al. 2011).

Despite documenting progress for women distinguished speakers, our study was unable to evaluate similar trends for nonbinary speakers. Out of our sample of 1692 speakers, only one identified as nonbinary. This observation reflects an overall pattern of limited research on the representation and inclusion of nonbinary scientists (Hall et al. 2022). Although there has been an increase in the use of gender-inclusive language, such as nonbinary pronouns, characteristics such as age and interest in gender issues influence their use (Gustafsson Sendén et al. 2021). Consequently, the widespread adoption of nonbinary pronouns in STEM fields and at ecology conferences may be delayed, because expectations of gender and sexuality remain heteronormative in many workplaces (Yoder and Mattheis 2016, Collins et al. 2024). Moreover, the frequent underreporting of nonbinary identities in STEM fields may be due to concerns about being outed (Langin 2020, Alexander et al. 2023). Changes in research and survey methods to better represent and protect nonbinary scientists are critical steps toward inclusion (Alexander et al. 2023). Increasing the inclusion and representation of nonbinary scientists will require intentional efforts to include such individuals in surveys, increase their representation in leadership positions (e.g., as distinguished speakers), and combat bullying and discrimination (Collins et al. 2024).

We recognize the limitation of our exclusive focus on gender in this study, which is one of many marginalized demographics. Unlike pronouns derived from a speaker's own biography, we could not accurately determine race, ethnicity, sexual orientation, or other characteristics of speakers who are associated with marginalized or excluded groups in ecology because speakers do not typically provide such information in their biographies. We highlight the need for future research to understand inclusion and representation of minoritized groups beyond gender identity or at the intersection of gender and other identities as distinguished speakers at professional conferences.

We also recognize a need to examine gender representation at teaching-focused conferences, particularly those attended by community college faculty. Because our study is largely focused on research-oriented societies, it remains unclear whether similar representation patterns exist in pedagogy-focused conferences that are attended more by ecologists in nonresearch positions. Investigating speaker selection in these settings could reveal unique barriers or trends not captured in our study.

## Addressing lingering gaps in gender representation

Improving gender representation across career stages, institutions, and fields within the ecology profession requires a multifaceted approach. Active measures should be implemented to combat gender inequalities in speaking roles, particularly in fields and institutions where women remain underrepresented. Such measures could include diversifying selection committees, establishing more equitable policies for selecting distinguished speakers and committee members, and striving for better representation of women in later career stages as distinguished speakers (Farr et al. 2017). Studies suggest that including women on conference planning committees significantly increases the proportion of women speakers represented at these events (Casadevall and Handelsman 2014, Arora et al. 2020). Societies should also accommodate needs associated with caregiving roles, which disproportionately fall on women. In addition, efforts should be made to increase the representation and inclusion of nonbinary speakers and to combat discrimination at conferences and beyond (Collins et al. 2024). Support groups within professional societies have been found to effectively support the well-being of nonbinary and other LGBTQIA+ scientists, alongside implementing policies and actions that offer tangible support rather than performative gestures (Collins et al. 2024). Adopting these comprehensive strategies can help professional societies move toward a more inclusive and equitable future for scientists of all genders, ensuring that everyone has the opportunity to be recognized in their fields, and to inspire the next generation.

## Supplementary Material

biaf063_Supplemental_File

## Author Biography

Sara Bombaci (sara.bombaci@colostate.edu) and Liba Pejchar are affiliated with the Department of Fish, Wildlife, and Conservation Biology and with the Graduate Degree Program in Ecology at Colorado State University, in Fort Collins, Colorado, in the United States. Cooper Farr is affiliated with the Conservation Science Program at Tracy Aviary, in Salt Lake City, Utah, in the United States. Kate D. Wilkins is affiliated with the Department of Field Conservation at the Denver Zoo Conservation Alliance, in Denver, Colorado, in the United States. Tessa Wittman is affiliated with the Ruckelshaus Institute, Haub School of Environment and Natural Resources, at the University of Wyoming, in Laramie, Wyoming, in the United States.

## References

[bib1] Alexander NB et al. 2023. Disparities, concerns, and recommendations for LGBTQ+ data collection within the biological sciences. BioScience 73: 258–260.

[bib2] Ali HN et al. 2021. An actionable anti-racism plan for geoscience organizations. Nature Communications 12: 3794. 10.1038/s41467-021-23936-w PMC8219696 34158472

[bib3] Arora A, Kaur Y, Dossa F, Nisenbaum R, Little D, Baxter NN. 2020. Proportion of female speakers at academic medical conferences across multiple specialties and regions. JAMA Network Open 3: e2018127. 32986107 10.1001/jamanetworkopen.2020.18127 PMC7522699

[bib4] Aubry LM, Laverty TM, Ma Z. 2021. Impacts of COVID-19 on ecology and evolutionary biology faculty in the United States. Ecological Applications 31: e2265. 33226725 10.1002/eap.2265 PMC7744888

[bib5] Baker M, Halberstam Y, Kroft K, Mas A, Messacar D. 2019. Pay Transparency and the Gender Gap. National Bureau of Economic Research.

[bib6] Bartoń K. 2023. MuMIn: Multi-model inference. R package version 1.47.5. R Foundation for Statistical Computing.

[bib7] Bates D, Mächler M, Bolker B, Walker S. 2015. Fitting linear mixed-effects models using lme4. Journal of Statistical Software 67: 1–48. 10.18637/jss.v067.i01.

[bib8] Breuning M, Fattore C, Ramos J, Scalera J. 2021. The great equalizer? Gender, parenting, and scholarly productivity during the global pandemic. PS: Political Science and Politics 54: 427–431.

[bib9] Burdett HL, Kelling I, Carrigan M. 2022. #TIMESUP: Tackling gender inequities in marine and fisheries science. Journal of Fish Biology 100: 4–9. 34724204 10.1111/jfb.14936

[bib10] Burnham KP, Anderson DR. 2002. Model Selection and Multimodel Inference: A Practical Information-Theoretic Approach, 2nd ed. Springer.

[bib11] Cardel MI, Dhurandhar E, Yarar-Fisher C, Foster M, Hidalgo B, McClure LA, Pagoto S, Brown N, Pekmezi D, Sharafeldin N. 2020. Turning chutes into ladders for women faculty: A review and roadmap for equity in academia. Journal of Women's Health 29: 721–733. 10.1089/jwh.2019.8027 PMC7247039 32043918

[bib12] Carli LL, Alawa L, Lee Y, Zhao B, Kim E. 2016. Stereotypes about gender and science: Women ≠ scientists. Psychology of Women Quarterly 40: 244–260.

[bib13] Casad BJ, Franks JE, Garasky CE, Kittleman MM, Roesler AC, Hall DY, Petzel ZW. 2021. Gender inequality in academia: Problems and solutions for women faculty in STEM. Journal of Neuroscience Research 99: 13–23. 33103281 10.1002/jnr.24631

[bib14] Casadevall A, Handelsman J. 2014. The presence of female conveners correlates with a higher proportion of female speakers at scientific symposia. MBio 5: e00846–13. 24399856 10.1128/mBio.00846-13 PMC3884059

[bib15] Catalán N et al. 2023. Women in limnology: From a historical perspective to a present-day evaluation. WIREs Water 10: e1616.

[bib16] Cech EA, Blair-Loy M. 2019. The changing career trajectories of new parents in STEM. Proceedings of the National Academy of Sciences 116: 4182–4187. 10.1073/pnas.1810862116 PMC6410805 30782835

[bib17] Collins A, Feuka A, Nelson J, Verahrami A, Bombaci SP. 2024. Perspectives on inclusion, safety, and belonging from members of the North American LGBTQIA+ conservation community. Conservation Biology 38: e14389. 39587021 10.1111/cobi.14389 PMC11589001

[bib18] Debarre F, Rode NO, Ugelvig LV. 2018. Gender equity at scientific events. Evolution Letters 2: 148–158. 30283672 10.1002/evl3.49 PMC6121837

[bib19] Dizney LJ, Karr J, Rowe RJ. 2019. The contribution and recognition of women in the field of mammalogy. Journal of Mammalogy 100: 678–689.

[bib20] Downing RA, Crosby FJ, Blake-Beard S. 2005. The perceived importance of developmental relationships on women undergraduates’ Pursuit of science. Psychology of Women Quarterly 29: 419–426.

[bib21] Drury BJ, Siy JO, Cheryan S. 2011. When do female role models benefit women? The importance of differentiating recruitment from retention in STEM. Psychological Inquiry 22: 265–269.

[bib22] Elisburg D. 1978. Equal pay in the United States: The development and implementation of the Equal Pay Act of 1963. Labor Law Journal 29: 195.

[bib23] Farr CM, Bombaci SP, Gallo T, Mangan AM, Riedl HL, Stinson LT, Wilkins K, Bennett DE, Nogeire-McRae T, Pejchar L. 2017. Addressing the gender gap in distinguished speakers at professional ecology conferences. BioScience 67: 464–468.

[bib24] Feldon DF, Peugh J, Maher MA, Roksa J, Tofel-Grehl C. 2017. Time-to-credit gender inequities of first-year PhD students in the biological sciences. CBE—Life Sciences Education 16: 4. 10.1187/cbe.16-08-0237 PMC5332047 28130271

[bib25] Fox J, Weisberg S. 2019. An R Companion to Applied Regression. Sage.

[bib26] Gardner SK. 2012. “I couldn't wait to leave the toxic environment”: A mixed methods study of women faculty satisfaction and departure from one research institution. NASPA Journal About Women in Higher Education 5: 71–95.

[bib27] Gavriilidi I, Van Damme R. 2023. Gender differences in animal cognition science. Animal Cognition 26: 1295–1305. 37071241 10.1007/s10071-023-01777-y

[bib28] Gustafsson Sendén M, Renström E, Lindqvist A. 2021. Beyond the binary: The change of attitudes and use over time. Gender and Society 35: 588–615.

[bib29] Hall CA et al. 2022. Diversifying the geosciences in higher education: A manifesto for change. Geoscience Communication 5: 275–280.

[bib30] Hartig F. 2022. DHARMa: Residual Diagnostics for Hierarchical (Multi-Level/Mixed) Regression Models. R Foundation for Statistical Computing. https://cran.r-project.org/web/packages/DHARMa/vignettes/DHARMa.html.

[bib31] Isbell LA, Young TP, Harcourt AH. 2012. Stag parties linger: Continued gender bias in a female-rich scientific discipline. PLOS ONE 7: e49682. 23185407 10.1371/journal.pone.0049682 PMC3504155

[bib32] Jensen AJ, Bombaci SP. 2024. Shaping scientists: How faculty values influence graduate student recruitment and diversity, equity, and inclusion. BioScience 74: 369–382. 10.1093/biosci/biae047

[bib33] Johnson R. 2013. The Importance of Social Movements and the Intersection of Social Equity: Marriage Equality and RACING towards Justice. Public and Nonprofit Administration.

[bib34] Joseph TD, Hirshfield LE. 2011. “Why don't you get somebody new to do it?” Race and cultural taxation in the academy. Ethnic and Racial Studies 34: 121–141.

[bib35] Joseph TD, Hirshfield LE. 2023. Reexamining racism, sexism, and identity taxation in the academy. Ethnic and Racial Studies 46: 1101–1108.

[bib36] Kalejta RF, Palmenberg AC. 2017. Gender parity trends for invited speakers at four prominent virology conference series. Journal of Virology 91: 10–1128. 10.1128/JVI.00739-17 PMC5533912 28592542

[bib37] Langin K. 2020. LGBTQ researchers say they want to be counted. Science 370: 1391–1391. 33335044 10.1126/science.370.6523.1391

[bib38] Legg S, Wang C, Kappel E, Thompson L. 2023. Gender equity in oceanography. Annual Review of Marine Science 15: 15–39. 10.1146/annurev-marine-032322-100357 35878677

[bib39] Lerman SB, Pejchar L, Benedict L, Covino KM, Dickinson JL, Fantle-Lepczyk JE, Rodewald AD, Vleck C. 2021. Juggling parenthood and ornithology: A full lifecycle approach to supporting mothers through the American Ornithological Society. Ornithological Applications 123: duab001.

[bib40] Light AE, Benson-Greenwald TM, Diekman AB. 2022. Gender representation cues labels of hard and soft sciences. Journal of Experimental Social Psychology 98: 104234.

[bib41] Louis WR. 2009. Collective action: And then what? Journal of Social Issues 65: 727–748.

[bib42] Ludecke D. 2023. sjPlot: Data visualization for statistics in Social science. R package version 2.8.15. R Foundation for Statistical Computing.

[bib43] Maple TL. 2021. The practice of management: The ascent of women as scholars and leaders in the field of zoo biology. Psychologist-Manager Journal 24: 97–114.

[bib44] Martin JL. 2014. Ten simple rules to achieve conference speaker gender balance. PLOS Computational Biology 10: e1003903. 25411977 10.1371/journal.pcbi.1003903 PMC4238945

[bib45] Martinez LR, O'Brien KR, Hebl MR. 2017. Fleeing the ivory tower: Gender differences in the turnover experiences of women faculty. Journal of Women's Health 26: 580–586. 10.1089/jwh.2016.6023 28437217

[bib46] McKeown M. 2021. Structural injustice. Philosophy Compass 16: e12757.

[bib47] Moeschler SM, Gali B, Goyal S, Schroeder DR, Jacobson J, Habermann EB, Keegan MT, Hyder JA. 2019. Speaker gender representation at the American Society of Anesthesiology Annual Meeting: 2011–2016. Anesthesia and Analgesia 129: 301. 30489314 10.1213/ANE.0000000000003944

[bib48] Niner HJ, Johri S, Meyer J, Wassermann SN. 2020. The pandemic push: Can COVID-19 reinvent conferences to models rooted in sustainability, equitability and inclusion? Socio-Ecological Practice Research 2: 253–256. 34765878 10.1007/s42532-020-00059-y PMC7446603

[bib49] Nittrouer CL, Hebl MR, Ashburn-Nardo L, Trump-Steele RCE, Lane DM, Valian V. 2018. Gender disparities in colloquium speakers at top universities. Proceedings of the National Academy of Sciences 115: 104–108. 10.1073/pnas.1708414115 PMC5776791 29255050

[bib50] Pell AN. 1996. Fixing the leaky pipeline: Women scientists in academia. Journal of Animal Science 74: 2843. 8923199 10.2527/1996.74112843x

[bib51] Potvin DA, Burdfield-Steel E, Potvin JM, Heap SM. 2018. Diversity begets diversity: A global perspective on gender equality in scientific society leadership. PLOS ONE 13: e0197280 29847591 10.1371/journal.pone.0197280 PMC5976142

[bib52] R Core Team . 2023. R: A Language and Environment for Statistical Computing. R Foundation for Statistical Computing.

[bib53] Rock KN, Barnes IN, Deyski MS, Glynn KA, Milstead BN, Rottenborn ME, Andre NS, Dekhtyar A, Dekhtyar O, Taylor EN. 2021. Quantifying the gender gap in authorship in herpetology. Herpetologica 77: 1–13.

[bib54] Ross MB, Glennon BM, Murciano-Goroff R, Berkes EG, Weinberg BA, Lane JI. 2022. Women are credited less in science than men. Nature 608: 135–145. 35732238 10.1038/s41586-022-04966-w PMC9352587

[bib55] Sarabipour S, Schwessinger B, Mumoki FN, Mwakilili AD, Khan A, Debat HJ, Sáez PJ, Seah S, Mestrovic T. 2020. Evaluating features of scientific conferences: A call for improvements. Nature Human Behavior 5: 296–300. 10.1038/s41562-021-01067-y 33723404

[bib56] Sardelis S, Drew JA. 2016. Not “pulling up the ladder”: Women who organize conference symposia provide greater opportunities for women to speak at conservation conferences. PLOS ONE 11: e0160015. 27467580 10.1371/journal.pone.0160015 PMC4965090

[bib57] Sardelis S, Oester S, Liboiron M. 2017. Ten strategies to reduce gender inequality at scientific conferences. Frontiers in Marine Science 4: 231.

[bib58] Sarsons H. 2017. Recognition for group work: Gender differences in academia. American Economic Review 107: 141–145.

[bib59] Shannon G, Jansen M, Williams K, Caceres C, Motta A, Odhiambo A, Eleveld A, Mannell J. 2019. Gender equality in science, medicine, and global health: Where are we at and why does it matter? Lancet 393: 560–569. 30739691 10.1016/S0140-6736(18)33135-0

[bib60] Shillcutt SK, Lorenzen KA. 2020. Whose voices are heard? Speaker gender representation at the Society of Cardiovascular Anesthesiologists annual meeting. Journal of Cardiothoracic and Vascular Anesthesia 34: 1805–1809. 32115361 10.1053/j.jvca.2020.01.046

[bib61] Singhal D, Bank AM, Poorman JA, Doshi TL, Parekh R, Parangi S, Hopf HW, Chandrabose R, Larson AR, Silver JK. 2020. Representation of women plenary speakers at the American Academy of Neurology Annual Meeting. Neurology 95: e3045–e3059. 33109622 10.1212/WNL.0000000000011058

[bib62] Singleton KS, Murray D-SRK, Dukes AJ, Richardson LNS. 2021. A year in review: Are diversity, equity, and inclusion initiatives fixing systemic barriers? Neuron 109: 3365–3367. 34358432 10.1016/j.neuron.2021.07.014

[bib63] Spoon K, LaBerge N, Wapman KH, Zhang S, Morgan AC, Galesic M, Fosdick BK, Larremore DB, Clauset A. 2023. Gender and retention patterns among U.S. faculty. Science Advances 9: eadi2205. 37862417 10.1126/sciadv.adi2205 PMC10588949

[bib64] Stefanova V, Farrell L, Latu I. 2023. Gender and the pandemic: Associations between caregiving, working from home, personal, and career outcomes for women and men. Current Psychology 42: 17395–17411. 10.1007/s12144-021-02630-6 PMC8717695 35002182

[bib65] Stepan-Norris J, Kerrissey J. 2016. Enhancing gender equity in academia: Lessons from the ADVANCE Program. Sociological Perspectives 59: 225–245.

[bib66] Stockard J, Rohlfing CM, Richmond GL. 2021. Equity for women and underrepresented minorities in STEM: Graduate experiences and career plans in chemistry. Proceedings of the National Academy of Sciences 118: e2020508118. 10.1073/pnas.2020508118 PMC7848687 33431653

[bib67] Thébaud S, Charles M. 2018. Segregation, stereotypes, and STEM. Social Sciences 7: 111.

[bib68] Toraif N, Gondal N, Paudel P, Frisellaa A. 2023. From colorblind to systemic racism: Emergence of a rhetorical shift in higher education discourse in response to the murder of George Floyd. PLOS ONE 18: e0289545. 37535657 10.1371/journal.pone.0289545 PMC10399877

[bib69] Uluğ ÖM, Acar YG. 2018. What happens after the protests? Understanding protest outcomes through multi-level social change: Peace and conflict. Journal of Peace Psychology 24: 44–53.

[bib70] Vallence et al. 2019. A data-driven approach to selecting invited speakers at conferences: A step toward gender parity. BioRxiv 426320. 10.1101/426320

[bib71] Vaughan K, Van Miegroet H, Pennino A, Pressler Y, Duball C, Brevik EC, Berhe AA, Olson C. 2019. Women in Soil science: Growing participation, emerging gaps, and the opportunities for advancement in the USA. Soil Science Society of America Journal 83: 1278–1289.

[bib72] Wapman KH, Zhang S, Clauset A, Larremore DB. 2022. Quantifying hierarchy and dynamics in US faculty hiring and retention. Nature 610: 120–127. 36131023 10.1038/s41586-022-05222-x PMC9534765

[bib73] Wickham H. 2016. ggplot2: Elegant Graphics for Data Analysis. Springer.

[bib74] Yoder JB, Mattheis A. 2016. Queer in STEM: Workplace experiences reported in a national survey of LGBTQA individuals in science, technology, engineering, and mathematics careers. Journal of Homosexuality 63: 1–27. 26241115 10.1080/00918369.2015.1078632

