# Supplementary material for: Forward steps, lingering gaps: gender representation among distinguished speakers at professional conferences
Source: Bioscience. 2025 Jun 30;75(9):737–46. doi: 10.1093/biosci/biaf063 (PMC12412295; doi:10.1093/biosci/biaf063)
Supplement: biaf063_Supplemental_File [file biaf063_supplemental_file.docx]

## Appendices

**Appendix S1 Table 1.** Number and percent of invited speakers at professional conferences in ecology who use she/her pronouns in each of the professional societies from 2000-2023 (n=1692 speakers, 249 conferences, and 70 societies including several combined societies). One speaker from the Society of American Foresters used they/them pronouns.

| **Professional Society** | **# She/her Speakers** | **Total # Speakers** | **Percent She/her** |
| --- | --- | --- | --- |
| American Fisheries Society | 32 | 95 | 34 |
| American Ornithological Society | 6 | 13 | 46 |
| American Ornithologists’ Union | 5 | 18 | 28 |
| American Society of Ichthyologists and Herpetologists | 10 | 26 | 38 |
| American Society of Limnology and Oceanography | 22 | 61 | 36 |
| American Society of Mammalogists | 44 | 130 | 34 |
| Animal Behavior Society | 46 | 96 | 48 |
| Aquatic Combined^a^ | 3 | 5 | 60 |
| Association for the Sciences of Limnology and Oceanography | 50 | 106 | 47 |
| Association of Field Ornithologists | 2 | 4 | 50 |
| Birds Combined^b^ | 55 | 125 | 44 |
| Botanical Society of America | 33 | 71 | 46 |
| Consortium of Aquatic Science Societies | 11 | 20 | 55 |
| Cooper Ornithological Society | 1 | 7 | 14 |
| Ecological Society of America | 38 | 85 | 45 |
| Ecology Combined^c^ | 3 | 7 | 43 |
| Entomological Society of America | 19 | 47 | 40 |
| Evolution Combined^d^ | 15 | 37 | 41 |
| Herptiles Combined^e^ | 5 | 34 | 15 |
| Marine Combined^f^ | 14 | 34 | 41 |
| Phycological Society of America | 9 | 23 | 39 |
| Range Combined^g^ | 1 | 7 | 14 |
| Raptor Research Foundation | 13 | 35 | 37 |
| Society for Conservation Biology | 39 | 96 | 41 |
| Society for Ecological Restoration | 19 | 34 | 56 |
| Society for Freshwater Science | 17 | 44 | 39 |
| Society for Integrative and Comparative Biology | 22 | 53 | 42 |
| Society for Range Management | 23 | 84 | 27 |
| Society for the Study of Evolution | 1 | 3 | 33 |
| Society for Urban Ecology | 1 | 5 | 20 |
| Society of American Foresters | 44 | 117 | 38 |
| Society of Wetland Scientists | 7 | 13 | 54 |
| The Wildlife Society | 23 | 69 | 33 |
| US-International Association of Landscape Ecology | 28 | 83 | 34 |
| Wilson Ornithological Society | 0 | 5 | 0 |

^a^Aquatic Combined: Society for Freshwater Science, Phycological Society of America, Society of Wetland Scientists

^b^Birds Combined: American Ornithological Society, American Ornithologists' Union , Birds Caribbean, British Ornithologists Union, Society of Canadian Ornithologists, Association of Field Ornithologists, Wilson Ornithological Society, Birds Caribbean, CIPAMEX, Neotropical Ornithological Society, Para la Naturaleza, the Waterbird Society , Cooper Ornithological Society, Sociedad de Ornitologia Neotropical, Mexican Ornithological Society, Raptor Research Foundation, Society for the Conservation and Study of Caribbean Birds

^c^Ecology Combined: Ecological Society of America, Canadian Society for Ecology and Evolution

^d^Evolution Combined: Society for the Study of Evolution, Society of Systemic Biologists, American Society of Naturalists, and European Society for Evolutionary Biology, Canadian Society for Ecology and Evolution, European Society for Evolutionary Biology

^e^Herptiles Combined: Society for the Study of Amphibians and Reptiles, American Society of Ichthyologists and Herpetologists, Partners in Amphibian and Reptile Conservation

^f^Marine Combined: American Geophysical Union, Association for the Sciences of Limnology and Oceanography, The Oceanography Society, American Society of Limnology and Oceanography, North American Benthological Society

^g^Range Combined: Society for Range Management, Weed Science Society of America, American Forage and Grassland Council

**Appendix S1 Table 2.** Subfield classifications (bold) for the ecological societies (listed below each bolded subfield) hosting the conferences.

| **Animal Behavior** |
| --- |
| Animal Behavior Society |
| **Botany/Plant Biology** |
| Botanical Society of America |
| Phycological Society of America |
| **Conservation biology** |
| Society for Conservation Biology |
| Entomology |
| Entomological Society of America |
| **Evolutionary Biology** |
| American Society of Naturalists |
| Canadian Society for Ecology and Evolution |
| European Society for Evolutionary Biology |
| Society for the Study of Evolution |
| Society of Systemic Biologists |
| **Fisheries** |
| American Fisheries Society |
| **Forestry Science** |
| Society of American Foresters |
| **General Ecology** |
| Canadian Botanical Association |
| Canadian Society for Ecology and Evolution |
| Ecological Society of America |
| Ecological Society of America and Canadian Society for Ecology and Evolution (CSEE) |
| Society for Freshwater Science |
| **General Fish and Wildlife** |
| The Wildlife Society |
| **Herpetology & Ichthyology** |
| American Society of Ichthyologists and Herpetologists |
| Partners in Amphibian and Reptile Conservation |
| Society for the Study of Amphibians and Reptiles |
| **Landscape Ecology** |
| Society of Wetland Scientists |
| US-International Association of Landscape Ecology |
| **Mammalogy** |
| American Society of Mammalogists |
| **Marine Biology and Biological Oceanography** |
| American Geophysical Union^a^ |
| American Society of Limnology and Oceanography |
| Association for the Sciences of Limnology and Oceanography |
| North American Benthological Society |
| Phycological Society of America |
| Society of Wetland Scientists |
| The Oceanography Society |
| Society for Freshwater Science |
| **Natural Resources/Conservation** |
| Society for Conservation Biology |
| **Ornithology** |
| American Ornithologists' Union |
| Association of Field Ornithologists |
| Birds Caribbean |
| British Ornithologists Union |
| CIPAMEX |
| Cooper Ornithological Society |
| Mexican Ornithological Society |
| Neotropical Ornithological Society |
| Para la Naturaleza |
| Raptor Research Foundation |
| Sociedad de Ornitologia Neotropical |
| Society for the Conservation and Study of Caribbean Birds |
| Society of Canadian Ornithologists |
| Waterbird Society |
| Wilson Ornithological Society |
| **Range Management** |
| American Forage and Grassland Council |
| Society for Range Management |
| Weed Science Society of America |
| **Restoration Ecology** |
| Society for Ecological Restoration |
| **Taxonomy/Comparative Biology** |
| Society for Integrative and Comparative Biology |
| **Urban Ecology** |
| Society for Urban Ecology |

^a^ The American Geophysical Union is under Marine Biology and Biological Oceanography because it co-hosted conferences focused on this topic with the other societies in this category.

**Appendix S1 Table 3.** Summary of categories of data collected for distinguished speakers invited to speak at professional conferences in ecology from 2016-2023.

| Society(ies) | (see Appendix S1 Table 1) |
| --- | --- |
| Conference | (see Appendix S1 Table 1) |
| Conference Subfield | (see Appendix S1 Table 2) |
| Year | 2016-2023 |
| Attendance Type | In-person; virtual; hybrid |
| Speaker Name |  |
| Career Stage | Early; Mid; Late |
| Ecological Subfield | (see Appendix S2 Table TW1) |
| Field of Expertise | Arts & Humanities; Life & Physical Sciences; Policy: Social Sciences |
| Talk Type | Award Recipient; Keynote; Moderator/Panelist; Plenary |
| Organization |  |
| Institution Type | Academia; Government; Private Sector; Museums/Zoos/Aquaria/NGOs |
| Pronouns | She/her; He/him; They/them |

**Appendix S1 Table 4.** Citations for the National Science Foundation (NSF) data on total number of graduate students in ecology and women graduate students (listed as “female” in the NSF tables) in ecology from 2000-2023.

| **Years** | **Suggested Citation** |
| --- | --- |
| 2000-2003 | National Science Foundation, Division of Science Resources Statistics, Graduate Students and Postdoctorates in Science and Engineering: Fall 2003, NSF 06-307, Project Officer, Julia Oliver (Arlington, VA 2006). |
| 2004-2009 | National Science Foundation, National Center for Science and Engineering Statistics. 2013. Graduate Students and Postdoctorates in Science and Engineering: Fall 2010. Detailed Statistical Tables NSF 13-314. Arlington, VA. Available at http://www.nsf.gov/statistics/nsf13314/. |
| 2010-2015 | National Science Foundation, National Center for Science and Engineering Statistics, Survey of Graduate Students and Postdoctorates in Science and Engineering, Fall 2015. http://ncsesdata.nsf.gov/gradpostdoc/. |
| 2016 | National Center for Science and Engineering Statistics, Survey of Graduate Students and Postdoctorates in Science and Engineering, Fall 2016. http://ncsesdata.nsf.gov/gradpostdoc/. |
| 2017 | National Center for Science and Engineering Statistics, Survey of Graduate Students and Postdoctorates in Science and Engineering, Fall 2017. http://ncsesdata.nsf.gov/gradpostdoc/. |
| 2018 | National Center for Science and Engineering Statistics, Survey of Graduate Students and Postdoctorates in Science and Engineering, Fall 2018. http://ncsesdata.nsf.gov/gradpostdoc/. |
| 2019 | National Center for Science and Engineering Statistics (NCSES). 2023. Survey of Graduate Students and Postdoctorates in Science and Engineering. NSF 21-318. Alexandria, VA: National Science Foundation. Available at https://ncses.nsf.gov/pubs/nsf23312. |
| 2020 | National Center for Science and Engineering Statistics (NCSES). 2023. Survey of Graduate Students and Postdoctorates in Science and Engineering. NSF 22-319. Alexandria, VA: National Science Foundation. Available at https://ncses.nsf.gov/pubs/nsf23312. |
| 2021 | National Center for Science and Engineering Statistics (NCSES). 2023. Survey of Graduate Students and Postdoctorates in Science and Engineering. NSF 23-312. Alexandria, VA: National Science Foundation. Available at https://ncses.nsf.gov/pubs/nsf23312. |

**Appendix S1 Table 5.** Number and percent of invited speakers at professional conferences in ecology who use she/her pronouns in each career stage from 2000-2023 (n=1692). Career stages were defined using titles and years of postdoctoral experience. Early-career professionals were speakers with titles that included “assistant” or “junior” or those with 0–7 years of postdoctoral experience. Intermediate-career professionals were speakers with titles that included “associate” or those with 8–14 years of postdoctoral experience. Late-career professionals were speakers with titles that included “full,” “director,” or “senior” or those with more than 15 years of postdoctoral experience. One early career stage speaker used they/them pronouns.

| **Career Stage** | **# She/her Speakers** | **Total # Speakers** | **Percent She/her** |
| --- | --- | --- | --- |
| Early | 134 | 240 | 56 |
| Intermediate | 95 | 172 | 55 |
| Late | 432 | 1280 | 34 |

**Appendix S1 Table 6**. Model selection table for all models in the analysis. The + symbol indicates that the relevant categorical variable was included in the model. Values for Intercept and Year are regression coefficients.

| **Intercept** | **Career stage** | **COVID** | **Field of expertise** | **Institution type** | **Year (scaled)** | **df** | **logLik** | **AICc** | **ΔAICc** | **weight** |
| --- | --- | --- | --- | --- | --- | --- | --- | --- | --- | --- |
| 0.60 | + |  | + |  | 0.41 | 8 | -1061.65 | 2139.40 | 0.00 | 0.47 |
| 0.65 | + | + | + |  | 0.38 | 9 | -1061.53 | 2141.20 | 1.79 | 0.19 |
| 0.65 | + |  | + | + | 0.41 | 12 | -1058.58 | 2141.30 | 1.96 | 0.18 |
| 0.70 | + | + | + | + | 0.38 | 13 | -1058.46 | 2143.10 | 3.76 | 0.07 |
| 0.15 | + |  |  |  | 0.41 | 5 | -1067.06 | 2144.10 | 4.77 | 0.04 |
| 0.13 | + |  |  | + | 0.41 | 9 | -1063.48 | 2145.10 | 5.69 | 0.03 |
| 0.22 | + | + |  |  | 0.38 | 6 | -1066.89 | 2145.80 | 6.45 | 0.02 |
| 0.19 | + | + |  | + | 0.38 | 10 | -1063.32 | 2146.80 | 7.40 | 0.01 |
| 1.16 | + | + | + |  |  | 8 | -1074.07 | 2164.20 | 24.85 | 0.00 |
| 1.20 | + | + | + | + |  | 12 | -1070.92 | 2166.00 | 26.64 | 0.00 |
| 0.69 | + | + |  |  |  | 5 | -1079.68 | 2169.40 | 30.02 | 0.00 |
| 0.66 | + | + |  | + |  | 9 | -1075.89 | 2169.90 | 30.50 | 0.00 |
| -0.07 |  |  | + |  | 0.47 | 6 | -1084.40 | 2180.80 | 41.47 | 0.00 |
| 0.02 |  | + | + |  | 0.43 | 7 | -1084.08 | 2182.20 | 42.85 | 0.00 |
| -0.49 |  |  |  |  | 0.48 | 3 | -1089.00 | 2184.00 | 44.63 | 0.00 |
| -0.03 |  |  | + | + | 0.48 | 10 | -1082.39 | 2184.90 | 45.53 | 0.00 |
| -0.39 |  | + |  |  | 0.43 | 4 | -1088.62 | 2185.30 | 45.89 | 0.00 |
| 0.06 |  | + | + | + | 0.43 | 11 | -1082.06 | 2186.30 | 46.90 | 0.00 |
| -0.52 |  |  |  | + | 0.48 | 7 | -1086.67 | 2187.40 | 48.04 | 0.00 |
| -0.42 |  | + |  | + | 0.43 | 8 | -1086.29 | 2188.70 | 49.28 | 0.00 |
| 0.91 | + |  | + |  |  | 7 | -1088.74 | 2191.50 | 52.16 | 0.00 |
| 0.94 | + |  | + | + |  | 11 | -1085.25 | 2192.70 | 53.27 | 0.00 |
| 0.32 | + |  |  | + |  | 8 | -1090.99 | 2198.10 | 58.68 | 0.00 |
| 0.35 | + |  |  |  |  | 4 | -1095.49 | 2199.00 | 59.62 | 0.00 |
| 0.55 |  | + | + |  |  | 6 | -1100.65 | 2213.40 | 73.98 | 0.00 |
| 0.08 |  | + |  |  |  | 3 | -1105.40 | 2216.80 | 77.43 | 0.00 |
| 0.57 |  | + | + | + |  | 10 | -1098.72 | 2217.60 | 78.20 | 0.00 |
| 0.05 |  | + |  | + |  | 7 | -1103.01 | 2220.10 | 80.72 | 0.00 |
| 0.10 |  |  | + |  |  | 5 | -1123.53 | 2257.10 | 117.72 | 0.00 |
| 0.12 |  |  | + | + |  | 9 | -1121.58 | 2261.30 | 121.88 | 0.00 |
| -0.46 |  |  |  |  |  | 2 | -1129.33 | 2262.70 | 123.29 | 0.00 |
| -0.51 |  |  |  | + |  | 6 | -1126.66 | 2265.40 | 125.99 | 0.00 |

**Appendix S1 Table 7.** Number and percent of invited speakers at professional conferences in ecology who use she/her pronouns in each talk type from 2016-2023 (n=883). One moderator/panelist used they/them pronouns

| **Talk Type** | **# She/her Speakers** | **Total # Speakers** | **Percent She/her** |
| --- | --- | --- | --- |
| Award Recipient | 121 | 297 | 41 |
| Keynote | 105 | 247 | 43 |
| Moderator/Panelist | 77 | 154 | 50 |
| Plenary | 358 | 994 | 36 |

**Appendix S1 Table 8.** Number and percent of invited speakers at professional conferences in ecology who use she/her pronouns in each field of expertise from 2000-2023 (n=1692). One speaker with expertise in social sciences used they/them pronouns.

| **Field of Expertise** | **# She/her Speakers** | **Total # Speakers** | **Percent She/her** |
| --- | --- | --- | --- |
| Arts and Humanities | 16 | 31 | 52 |
| Life and Physical Sciences | 563 | 1491 | 38 |
| Policy | 32 | 73 | 44 |
| Social Sciences | 50 | 97 | 52 |
|  |  |  |  |

**Appendix S1 Table 9.** Number and percent of invited speakers at professional conferences in ecology who use she/her pronouns in each of the institution types from 2000-2023 (n=1692). One speaker from academia used they/them pronouns. Museums/Zoo/Aquaria/NGOs also includes botanic gardens.

| **Institution Type** | **# She/her Speakers** | **Total # Speakers** | **Percent She/her** |
| --- | --- | --- | --- |
| Academia | 434 | 1135 | 38 |
| Government | 83 | 226 | 37 |
| Private Sector | 36 | 93 | 39 |
| Museums/Zoos/Aquaria/NGOs | 101 | 220 | 46 |
| Tribal | 7 | 18 | 39 |

**Appendix S1 Table 10.** Number and percent of invited speakers at professional conferences in ecology who use she/her pronouns in each of the ecological subfields from 2000-2023 (n=1692). One speaker in forestry science used they/them pronouns.

| **Ecological Subfield** | **# She/her Speakers** | **Total # speakers** | **Percent She/her** |
| --- | --- | --- | --- |
| Animal Behavior | 46 | 96 | 48 |
| Botany and Plant Biology | 42 | 94 | 45 |
| Conservation Biology | 39 | 96 | 41 |
| Entomology | 19 | 47 | 40 |
| Evolutionary Biology | 9 | 29 | 31 |
| Fish and Wildlife Biology | 55 | 164 | 34 |
| Forestry Science | 44 | 117 | 38 |
| General Ecology | 65 | 147 | 48 |
| Herpetology & Ichthyology^a^ | 15 | 60 | 25 |
| Landscape Ecology | 35 | 96 | 36 |
| Mammalogy | 44 | 130 | 34 |
| Marine Biology and Biological Oceanography | 100 | 226 | 44 |
| Ornithology | 82 | 207 | 40 |
| Range Management | 24 | 91 | 26 |
| Restoration Ecology | 19 | 34 | 56 |
| Taxonomy and Comparative Biology | 22 | 53 | 42 |
| Urban Ecology | 1 | 5 | 20 |

^a^Subfields were grouped when a majority of their conferences were held as joint meetings.

**
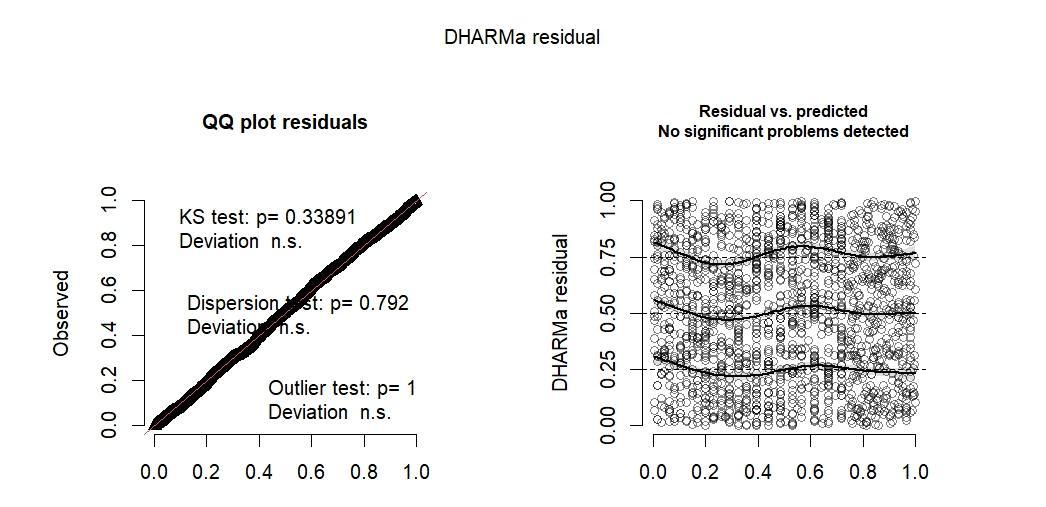
**

**
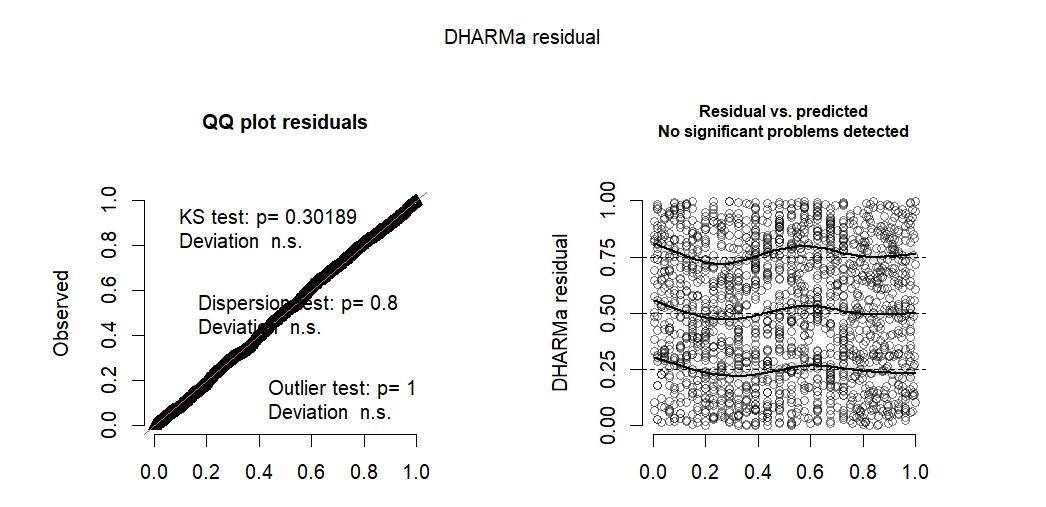
**

**
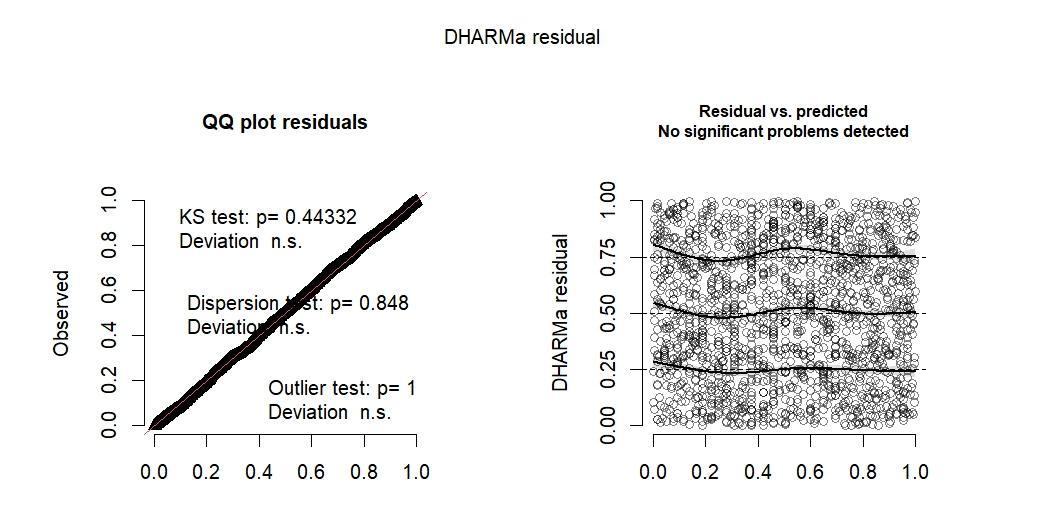
**

**
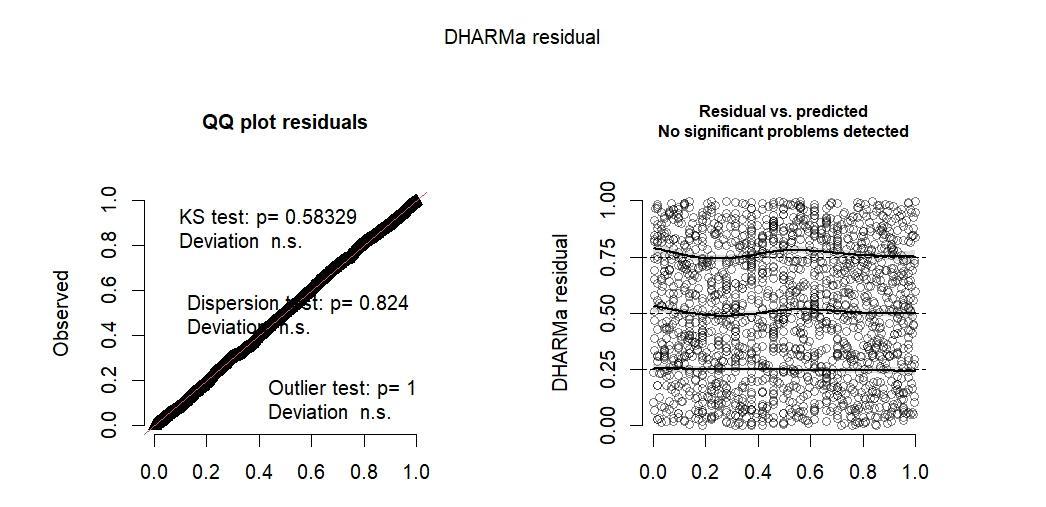
**

**Appendix S1 Figure 1.** Diagnostic plots from the top model (A), and second (B), third (C), and fourth (D) best supported models (all ΔAICc < 4).


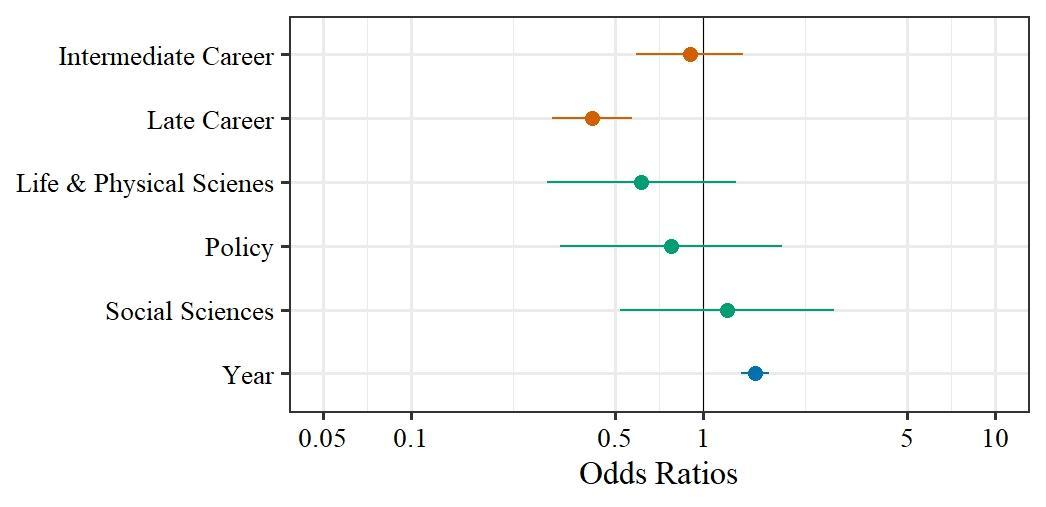


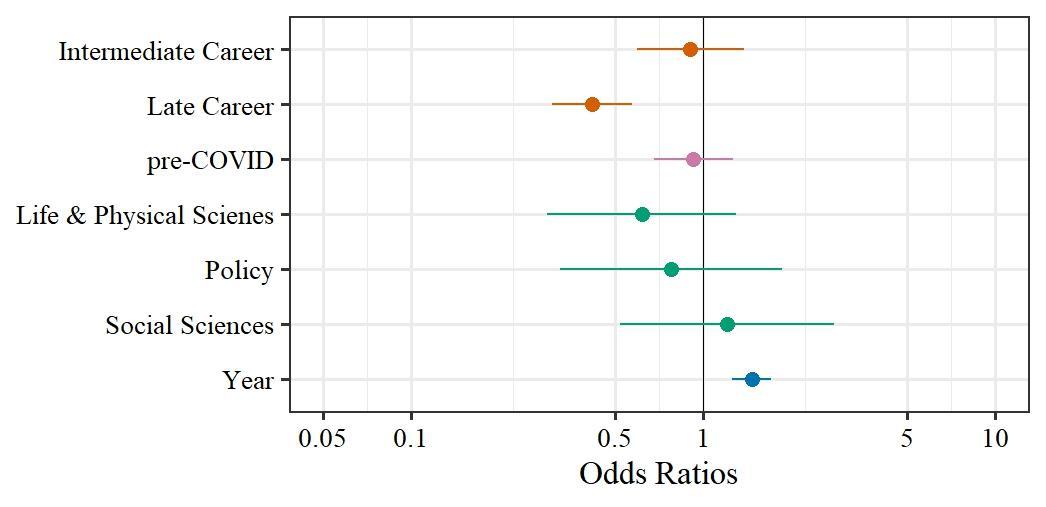

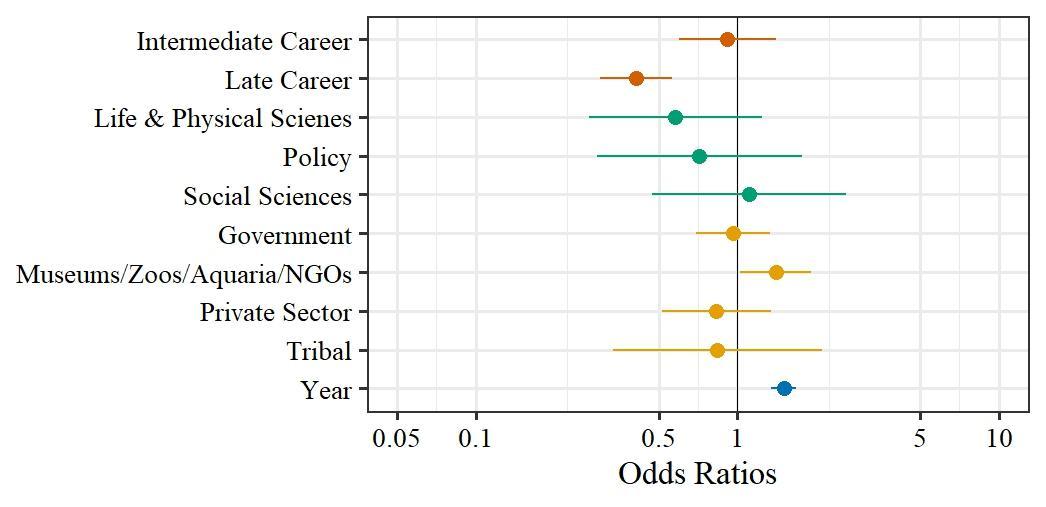


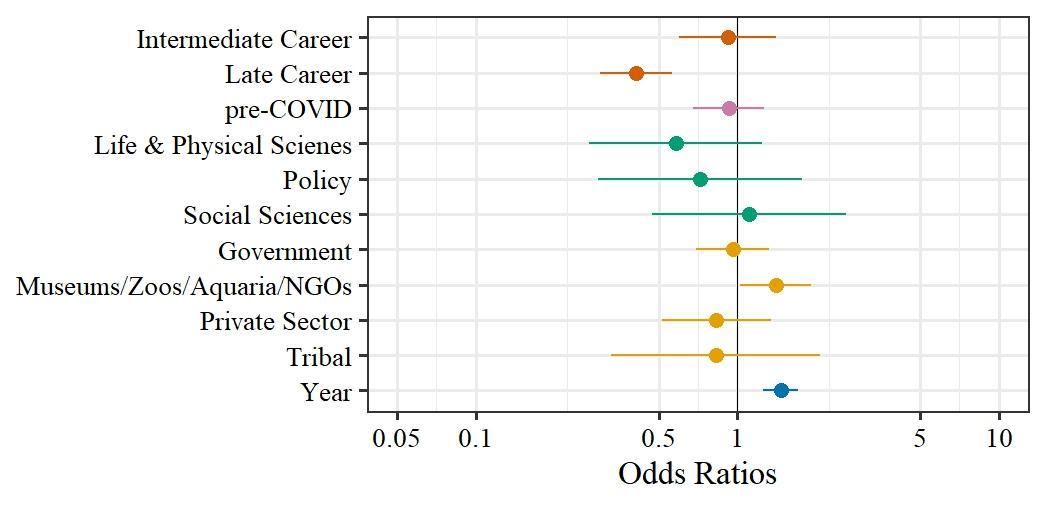


**Appendix S1 Figure 2.** Odd ratios and 95% confidence intervals from all four top models (delta AICc < 4) of characteristics that influenced the probability of a distinguished speaker being a woman. Odds ratios are for variables of career stage (early, intermediate, and late; orange), COVID (pre- or post-COVID; pink), field of expertise (arts and humanities, life and physical sciences, policy, and social sciences; green), institution type (academia, government, museums/zoos/aquaria/NGOs, private sector, tribal; yellow), and year (blue). Model order from top to bottom: best supported to fourth best supported model. An odds ratio of one indicates that the characteristic is equally likely to influence the probability of a speaker being a woman or a man. Odds ratios where 95% confidence intervals do not overlap one indicate that the characteristic is more likely to either positively or negatively influence the probability of a speaker being a woman.


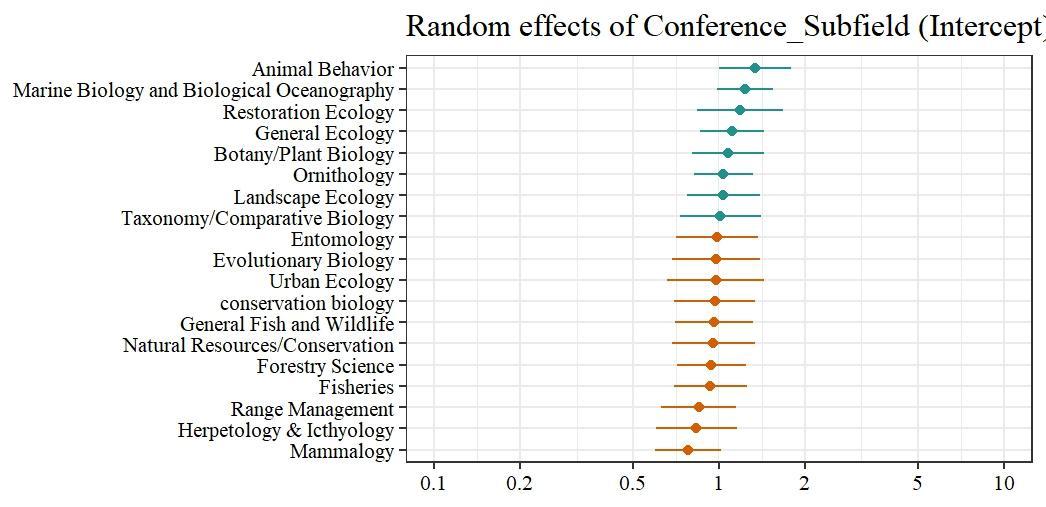


**Appendix S1 Figure 3.** Random effect coefficient values for the ‘conference subfield’ variable from the best supported model of the probability of a distinguished speaker being a woman. Values were similar across all four well-supported models.

**
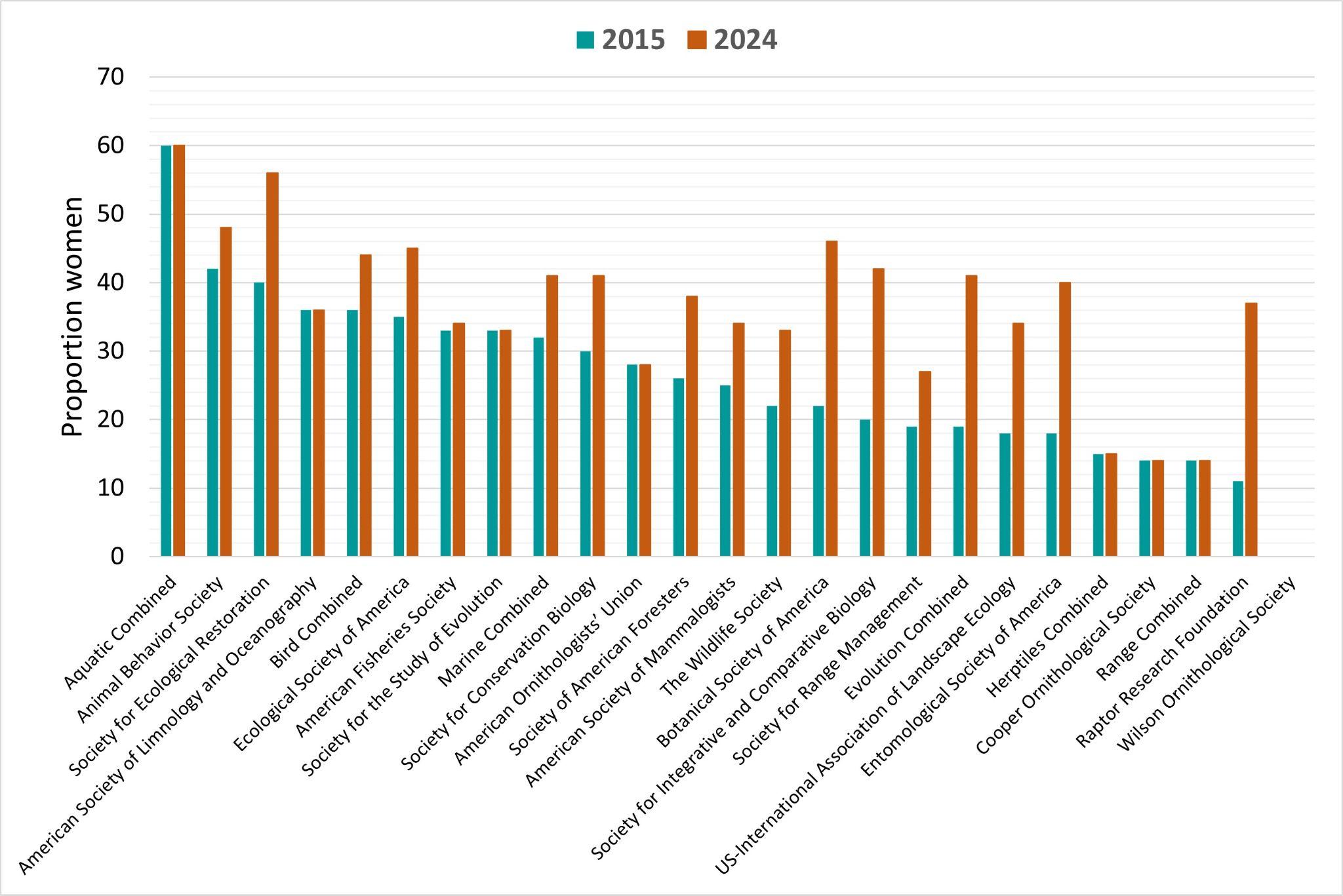
**

**Appendix S1 Figure 4.** Comparison of the proportion of women speakers for different professional societies between 2015 (Farr et al. 2017) and 2024 (current study). The Wilson Ornithological Society value is zero for both time periods.
